# Behavioral defects and downregulation of hippocampal BDNF and nNOS expression in db/db mice did not improved by chronic TGF-β2 treatment

**DOI:** 10.3389/fphys.2022.969480

**Published:** 2022-08-25

**Authors:** Yuki Tomiga, Yasuki Higaki, Keizo Anzai, Hirokazu Takahashi

**Affiliations:** ^1^ Division of Metabolism and Endocrinology, Faculty of Medicine, Saga University, Saga, Japan; ^2^ Japan Society for the Promotion of Science, Tokyo, Japan; ^3^ Fukuoka University Institute for Physical Activity, Fukuoka University, Fukuoka, Japan; ^4^ Faculty of Sports and Health Science, Fukuoka University, Fukuoka, Japan; ^5^ Liver Center, Saga University Hospital, Saga, Japan

**Keywords:** type 2 diabetes, BDNF (brain derived neurotrophic factor), hippocampus, TGF-β2, anxiety

## Abstract

Epidemiological evidence suggests that there is a link between diabetes and mood disorders, such as depression and anxiety. Although peripheral or central inflammation may explain this link, the molecular mechanisms are not fully understood and few effective treatments for diabetes or mood disorders are available. In the present study, we aimed to determine whether transforming growth factor (TGF)-β2, an anti-inflammatory substance, might represent a potential therapeutic agent for diabetes-related mood behaviors. TGF-β2 expression in the hippocampus is affected by anxiolytic drugs and stress exposure, it is able to cross the blood-brain barrier, and it is as an exercise-induced physiological adipokine that regulates glucose homeostasis. Therefore, we hypothesized that a chronic TGF-β2 infusion would ameliorate diabetes-related glucose intolerance and mood dysregulation. To determine the effects of the chronic administration of TGF-β2 on diabetes, we implanted osmotic pumps containing TGF-β2 into type 2 diabetic mice (*db/db* mice), and age-matched non-diabetic control wild type mice and *db/db* mice were infused with vehicle (PBS), for 12 consecutive days. To assess anxiety-like behaviors and glucose homeostasis, the mice underwent elevated plus maze testing and intraperitoneal glucose tolerance testing. Hippocampal and perigonadal visceral white adipose tissue perigonadal white adipose tissue samples were obtained 12 days later. Contrary to our hypothesis, TGF-β2 infusion had no effect on diabetes-related glucose intolerance or diabetes-related behavioral defects, such as inactivity. In *db/db* mice, the expression of inflammatory markers was high in pgWAT, but not in the hippocampus, and the former was ameliorated by TGF-β2 infusion. The expression of brain-derived neurotrophic factor and neuronal nitric oxide synthase, important regulators of anxiety-like behaviors, was low in *db/db* mice, but TGF-β2 infusion did not affect their expression. We conclude that although TGF-β2 reduces the expression of pro-inflammatory markers in the adipose tissue of diabetic mice, it does not ameliorate their obesity or mood dysregulation.

## Introduction

Obesity is becoming increasingly prevalent worldwide and predisposes people toward systemic metabolic diseases, such as type 2 diabetes (T2D). T2D is associated with complications in both the peripheral and central nervous system (CNS) (McCrimmon et al., 2012). In particular, epidemiological evidence has suggested that there is a close link between T2D and mood disorders. Being overweight, even in the absence of T2D, is associated with low hippocampal volume, which is important for mood regulation (Cherbuin et al., 2015). Patients with T2D have high incidences of several anxiety and affective disorders (7–123% higher than those of healthy, community-dwelling adults) (Fisher et al., 2008), and mood disorders, such as major depressive disorder (MDD), are present in a quarter of people with T2D (Semenkovich et al., 2015). However, the mechanisms underlying diabetes-related mood behaviors are still poorly understood, because of the complex bidirectional relationship between these disorders, and there are few effective treatments (Semenkovich et al., 2015).

Animal studies have shown that *db/db* mice, which a model of T2D, show abnormal behaviors, including depression-like or anxiety-like behaviors (Dinel et al., 2011; Liu et al., 2017; Fourrier et al., 2019; Zhang et al., 2019). Brain-derived neurotrophic factor (BDNF) in the hippocampus is important for mood regulation (Hashimoto et al., 2004) and its expression is low in *db/db* mice (Dinel et al., 2011; Shima et al., 2017; Wosiski-Kuhn et al., 2018). It has been shown that hippocampal BDNF expression is regulated by molecules including neuronal nitric oxide synthase (nNOS, encoded by *Nos1*) in the hippocampus (Stanquini et al., 2018). The results of several previous studies have suggested that peripheral or central inflammation may explain the low BDNF expression in T2D. Chronic inflammation, involving high serum tumor necrosis factor *α* (TNF-α) concentrations, accompanies large subcutaneous and visceral adipocytes (Winkler et al., 2003), and is associated with insulin resistance and T2D in both humans and rodents (Coppack, 2001; Lumeng and Saltiel, 2011; Wada et al., 2013). However, in the CNS, including the hippocampus, there have been conflicting findings regarding the expression of pro-inflammatory markers, such as TNF-α and interleukin (IL)-6 in *db/db* and diet-induced obese mice (Lavin et al., 2009; Dinel et al., 2011; Liu et al., 2017; Fourrier et al., 2019).

Transforming growth factor (TGF)-β is a widely expressed multifunctional growth factor that is also anti-inflammatory (Yoshimura et al., 2010). Three isoforms (TGF-β1, TGF-β2, and TGF-β3) have been identified in mammals. All three are present in the CNS, and TGF-β2 is the most abundant. Notably, the mRNA expression of TGF-β2 is higher in the hippocampus than in the cortex, striatum, brain stem, and cerebellum (Unsicker et al., 1991). Hippocampal *Tgfb2* is an anxiolytic drug-responsive gene (Lee et al., 2010): its expression is increased by anxiolytic drugs (Lee et al., 2010) and reduced by chronic, mild, unpredictable stress in a mouse model of depression (Grassi et al., 2017). In hippocampal neurons, TGF-β2 also acutely regulates synaptic plasticity and activates cAMP response element-binding protein (CREB) (Fukushima et al., 2007), an upstream regulator of BDNF (Conti et al., 2002). In a recent study, it was shown that TGF-β2 is a physical exercise-induced adipokine that improves the glucose homeostasis of high-fat diet (HFD)-fed obese mice (Takahashi et al., 2019). TGF-β2, but not TGF-β1, can cross the blood-brain barrier (Kastin et al., 2003; McLennan et al., 2005); thus, circulating TGF-β2 can affect the peripheral nervous system and directly access the CNS. Therefore, in the present study, we aimed to determine whether chronic TGF-β2 infusion ameliorates the glucose intolerance and behavioral deficits of *db/db* mice. In addition, we aimed to determine the effects of TGF-β2 infusion on hippocampal BDNF, its upstream molecules, and the expression of genes encoding pro-inflammatory proteins in *db/db* mice.

## Materials and methods

### Animals

Five-week-old male *db/db* (BKS.Cg-m+/+Lepr^*db*^/Jcl) and age-matched control wild type (WT) mice were purchased from CLEA (Tokyo, Japan). The number of mice used was determined by reference to previous studies (Bruchas et al., 2009; Tomiga et al., 2020). The mice were housed in an accredited animal facility that was maintained at a constant temperature (23.8 ± 0.2°C) and humidity (50.4 ± 1.9%), with a 12-h light/dark cycle, and were provided food and water *ad libitum*. The experiments were approved by the Saga University Animal Care and Use Committee (approval number: 1707075) and conducted in accordance with the regulations on animal experimentation at Saga University.

### Osmotic pump infusion of TGF-β2 *in vivo*

After 1 week of acclimatization, two mice were placed into each cage, to avoid social isolation, and cage-mates were not changed during the experiment. Chronic TGF-β2 infusion was performed as previously described (Takahashi et al., 2019). Briefly, the animals were randomly allocated to three groups at 10 weeks of age: WT + vehicle; *db/db* + vehicle, and *db/db* + TGF-β2 groups. Under isoflurane anesthesia (induction: 4%, maintenance: 2%), the mice underwent surgery to implant Alzet mini-osmotic pumps (model 1002, Durect, Cupertino, CA, United States) subcutaneously. The osmotic pumps were filled with recombinant TGF-β2 (8406LC, Cell Signaling Technologies, Danvers, MA, United States) diluted in PBS, or PBS alone for the WT + vehicle and *db/db* + vehicle groups. For 12 consecutive days, the mice received 12 ng TGF-β2 each per hour. After euthanasia, the pumps were removed and the contents were confirmed to have been completely discharged. The dose and duration of treatment were those used in a previous study (Takahashi et al., 2019).

### Behavioral testing

We assessed anxiety-like behaviors using the elevated plus maze (EPM) test, as previously described (Tomiga et al., 2021). The mice were transferred to the testing room a minimum of 60 min prior to each testing session. All the tests were performed between 11:00 and 14:00, during the light period (80–100 lux). Before each test, the testing apparatus was thoroughly cleaned with 70% (v/v) ethanol and dried to reduce olfactory cues. Briefly, the maze consisted of four arms (each arm was 30-cm long and 5-cm wide) and was placed 40 cm above the floor. Two arms contained side and end walls that were 15 cm high (the closed arms), and the other two arms had no walls (the open arms). The behavior of the mice was recorded using a video camera. The mice were placed in the center of the maze, facing an open arm, and were then allowed to explore for 5 min. Each arm entry, the resting time, and the time spent on slow and fast movement were analyzed using Smart 3.0 software (Panlab, Barcelona, Spain). The movement speed thresholds were defined as 1) rest: < 2.50 cm/s; 2) slow movement: 2.50–15.00 cm/s; 3) fast movement: >15.00 cm/s.

### Glucose tolerance testing (GTT)

The mice were fasted for 15–16 h, with free access to drinking water. Baseline blood samples were collected from the tails of fully conscious mice, which were then intraperitoneally administered glucose (1 g/kg). Fifteen, 30, 60, and 120 min later, blood samples were collected from the tail and their glucose concentrations were measured using a glucose meter (Stat Strip XP3, Nipro, Osaka, Japan).

### Tissue collection and weighing

The mice were euthanized at 12 weeks of age under isoflurane anesthesia. Blood samples were obtained and serum was isolated by centrifugation and frozen at −80°C until analyzed. The hippocampus, perigonadal white adipose tissue (pgWAT), and subcutaneous white adipose tissue (scWAT) were rapidly collected, and the tissues were placed in a tube containing RNA stabilization solution (Thermo Fisher Scientific, Waltham, MA, United States). The hippocampus and adipose tissue depots were weighed using an analytical balance. The mass of the tube plus the RNA stabilization solution was measured in advance, and the hippocampal mass was calculated by subtraction. Adipose tissue depots were rapidly weighed, and then portions of the pgWAT were immersed in RNA stabilization solution. After overnight incubation, the tissues were stored at −80°C for subsequent analysis. Because the mice were housed in pairs, total food intake was calculated in grams per cage per day (WT + vehicle: 6.085 ± 0.305 g/cage/day; *db/db* + vehicle: 11.79 ± 0.025 g/cage/day; and *db/db* + TGF-β2: 12.42 ± 0.29 g/cage/day.)

### Serum insulin analysis

Serum insulin concentration was measured using an ELISA kit (Morinaga, Kanagawa, Japan), according to the manufacturer’s instructions.

### Western blot

Protein lysates were prepared as described previously and aliquots containing 10 µg protein were separated by electrophoresis on a 14.0% (w/v) sodium dodecyl sulfate polyacrylamide gel and transferred to polyvinylidene fluoride membrane (Millipore, Billerica, MA, United States) using the semi-dry method (Tomiga et al., 2021). After transfer, Ponceau S staining (Beacle, Inc., Kyoto, Japan) was used to verify consistent loading, and the membrane was blocked with 3% (w/v) skim milk at room temperature for 1 h. The membrane was then incubated overnight at 4°C with the following primary antibodies: anti-BDNF (1:1,000; Abcam, ab108319; Cambridge, United Kingdom), which detects the precursor (proBDNF; 32 kDa) and mature (15 kDa) forms of BDNF, and anti-nNOS (#611852, BD Biosciences, San Jose, CA, United States). The membrane was then incubated with horseradish peroxidase-conjugated secondary antibodies (Vector Laboratories, Burlingame, CA, United States) for 1 h. The bound antibodies were detected using SuperSignal™ West Pico Plus Chemiluminescent Substrate (Thermo Fisher Scientific) and analyzed using the Fusion-FX7 imaging system (Vilber Lourmat, Marne-la-Vallée, France). Band densities were determined using ImageJ software (NIH, Bethesda, MD, United States) and the Ponceau S signal intensity was used as a loading control.

### Gene expression analysis

Hippocampal tissue and pgWAT were homogenized using a tissue homogenizer. RNA was extracted from the samples using a FastGene RNA Basic Kit (Nippon Gene, Tokyo, Japan). and assessed for concentration and purity using a NanoDrop 2000 (Thermo Fisher Scientific). The isolated RNA was then reverse transcribed to cDNA using PrimeScript RT Master Mix (Takara Bio, Otsu, Japan). qRT-PCR analyses were then performed using the QuantStudio 3 Real-Time PCR system (Applied Biosystems, Foster City, CA, United States). The mRNA expression of *Nos1a* and *Bdnf* (coding region, exon IX) was quantified using SYBR Green Master Mix (Applied Biosystems). The brain and pgWAT target gene expression was normalized to that of *Gapdh* or *Arbp* mRNA and quantified using the ΔΔCt method. All the samples were analyzed in duplicate. The primer sequences are listed in Table 1.

**TABLE 1:** Primers used for gene expression analysis.

Gene	Sequence 5′–3′
*Nos1α* reverse	TCA​AGG​TTG​ACC​AGG​CAG​ACG
*Bdnf* forward	TGG​CCC​TGC​GGA​GGC​TAA​GT
*Bdnf* reverse	AGG​GTG​CTT​CCG​AGC​CTT​CCT
*Tnfa* forward	CCA​CCA​CGC​TCT​TCT​GTC​T
*Tnfa* reverse	GCT​CCT​CCA​CTT​GGT​GGT​TT
*Il6* forward	TGA​TGC​ACT​TGC​AGA​AAA​CA
*Il6* reverse	GGT​ACT​CCA​GAA​GAC​CAG​AGG​A
*Tgfb2* forward	TTG​TTG​CCC​TCC​TAC​AGA​CTG​G
*Tgfb2* reverse	GTA​AAG​AGG​GCG​AAG​GCA​GCA​A
*Gapdh* forward	AACGACCCCTTCATTGAC
*Gapdh* reverse	TCC​ACG​ACA​TAC​TCA​GCA​C
*Arbp* forward	CGG​CCA​CGA​ACC​TCT​GTA​G
*Arbp* reverse	CTCATCCCCTGCCTTTGC

### Statistics

Data are presented as the mean ± standard error (SE). Statistical analyses were performed using Prism version 7.0 (GraphPad, San Diego, CA, United States). One-way ANOVA, followed by Bonferroni’s *post-hoc* test, was used to compare groups. Non-parametric datasets were compared using the Kruskal–Wallis test. Relationships between continuous variables were evaluated using Pearson’s product-moment correlations. *p* < 0.05 was considered to represent statistical significance.

## Results

### TGF-β2 infusion does not affect the body mass gain or glucose tolerance of *db/db* mice

**FIGURE 1:**
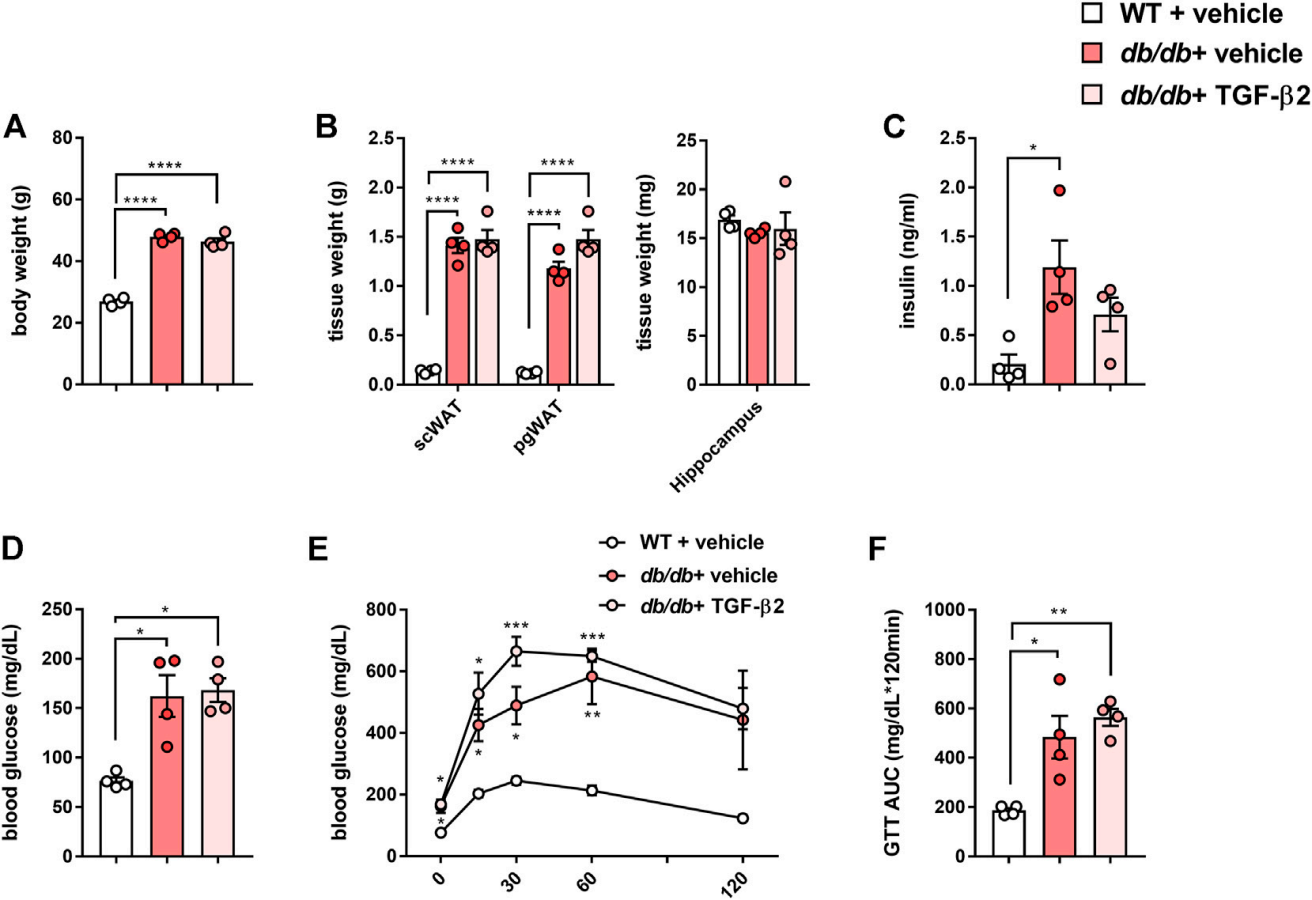
TGF-β2 infusion has no effects on the body mass gain or glucose tolerance of *db/db* mice **(A)** Body mass **(B)** scWAT, pgWAT, and hippocampal masses, **(C)** fasting serum insulin concentration **(D)** blood glucose concentration, **(E)** results of glucose tolerance testing, and **(F)** area under the glucose curve (AUC) for WT, *db/db* + vehicle, and *db/db* + TGF-β2 mice. Data are presented as the mean ± SE (*n* = 4/group). The dots represent individual data points. ****p < 0.0001; ***p < 0.001; **p < 0.01; *p < 0.05.

The body and adipose tissue masses of the *db/db* mice were significantly higher than those of the WT mice, but their hippocampal masses were similar (Figures 1A,B). In addition, the *db/db* mice had high fasting serum insulin and blood glucose concentrations and lower glucose tolerance than the WT mice (Figures 1C–F). TGF-β2 administration did not affect these parameters (Figure 1).

### TGF-β2 infusion has no effect on the low activity of *db/db* mice

**FIGURE 2:**
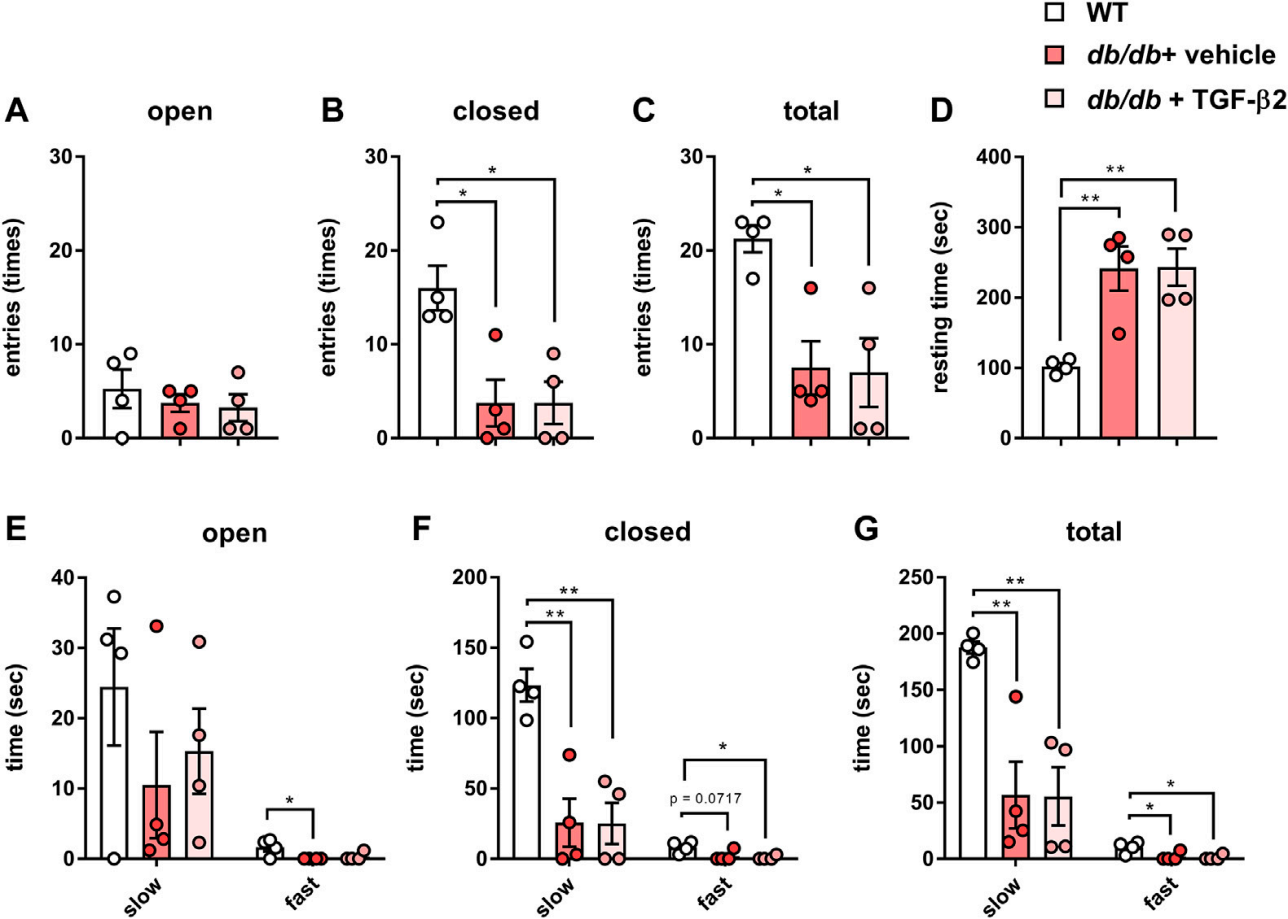
TGF-β2 infusion has no effect on the low locomotor activity of *db/db* mice. Numbers of **(A)** open, **(B)** closed, and **(C)** total arm entries **(D)** Resting time and time spent on slow and fast movement in the **(E)** open arms, **(F)** closed arms, and **(G)** both open and closed arms for the *db/db* + vehicle and *db/db* + TGF-β2 mice. Data are presented as the mean ± SE (*n* = 4/group). The dots represent individual data points. **p < 0.01; *p < 0.05.

To characterize the behavior of the *db/db* mice, we performed EPM testing. Although there was no difference in the number of open arm entries (Figure 2A), there were significantly fewer closed arm entries (Figure 2B). This smaller number of closed arm entries implies less anxiety-like behavior, but the total number of open and closed arm entries was also lower in the *db/db* mice (Figure 2C). In addition, the *db/db* mice spent more time resting during EPM testing than the WT mice (Figure 2D). Next, we analyzed the speed of movement of the mice in both the open and closed arms. With respect to the open arms, there was no difference in the time spent on slow movement among the groups, but the *db/db* mice spent less time moving rapidly (Figure 2E). With respect to the closed arms, the *db/db* mice spent less time on both slow and fast movement (Figure 2F). With respect to the combination of the open and closed arms, the *db/db* mice spent less time moving both slowly and rapidly (Figure 2G). However, there were no differences in these parameters between the *db/db +* TGF-β2 group and the *db/db* + vehicle group. Thus, the low level of locomotor activity of the diabetic mice was not ameliorated by TGF-β2 infusion.

### TGF-β2 infusion reduces pro-inflammatory marker expression in the pgWAT but not in the hippocampus of *db/db* mice

**FIGURE 3:**
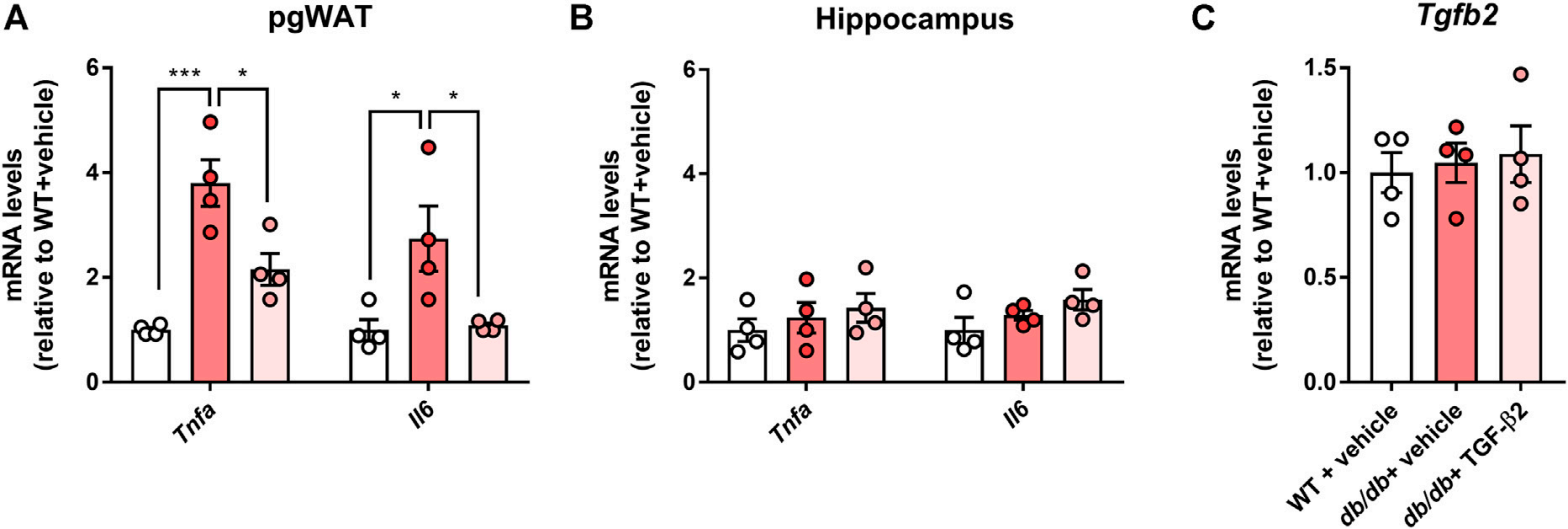
TGF-β2 infusion reduces the expression of genes encoding pro-inflammatory molecules in pgWAT, but not in the hippocampus, of *db/db* mice. *Tnfa* and *Il6* mRNA expression in the **(A)** pgWAT and **(B)** hippocampus **(C)** Hippocampal *Tgfb2* mRNA expression in WT, *db/db* + vehicle, and *db/db* + TGF-β2 mice. Data are presented as the mean ± SE (*n* = 4/group). The dots represent individual data points. ***p < 0.001; *p < 0.05.

In pgWAT, the expression of genes encoding pro-inflammatory molecules was significantly higher in the *db/db* + vehicle group than in the control group; however, this expression was lower in the *db/db* + TGF-β2 group (Figure 3A). There were no differences in the expression of genes encoding pro-inflammatory molecules in the hippocampus among the groups (Figure 3B). Hippocampal *Tgfb2* mRNA expression was also unaffected by the *db/db* genotype or the TGF-β2 infusion (Figure 3C).

### TGF-β2 infusion does not alter the low BDNF and nNOS protein and mRNA expression of *db/db* mice

**FIGURE 4:**
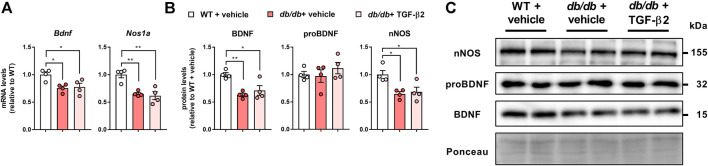
TGF-β2 infusion does not affect the low mRNA and protein expression of BDNF and nNOS in *db/db* mice **(A)** *Bdnf* and *Nos1a* mRNA expression, and **(B)** representative immunoblots and **(C)** quantification of BDNF, proBDNF, and nNOS protein levels in the hippocampi of WT, *db/db* + vehicle, and *db/db* + TGF-β2 mice. The Ponceau S signal intensity was used as a loading control for western blot analysis. Data are presented as the mean ± SE (*n* = 4/group). The dots represent individual data points. **p < 0.01; *p < 0.05.

**FIGURE 5:**
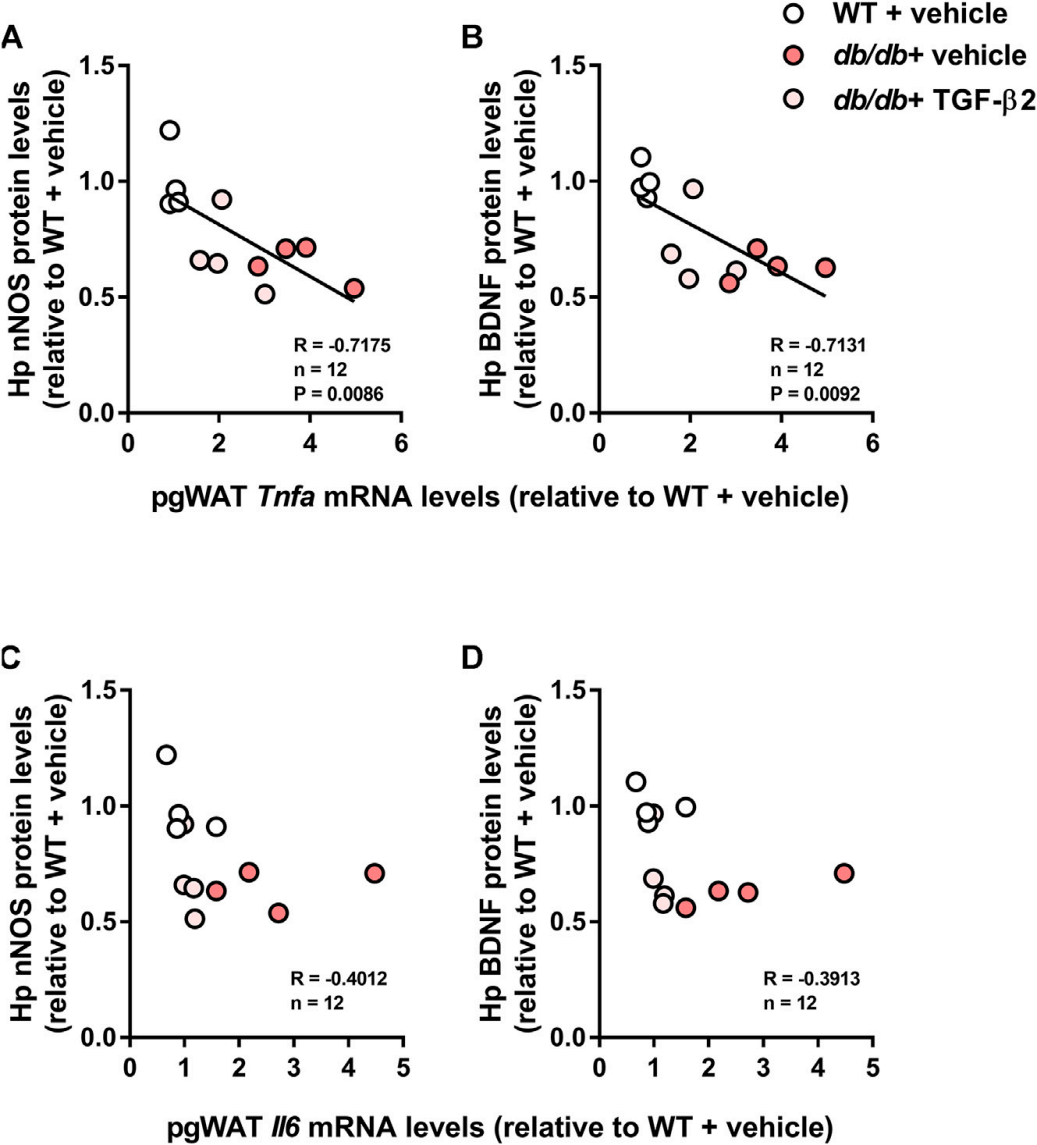
Hippocampal BDNF and nNOS protein levels correlate with pgWAT *Tnfa* mRNA expression, but not *Il6* mRNA expression, in WT, *db/db*, and TGF-β2 infused *db/db* mice. Correlations of pgWAT *Tnfa* mRNA expression with **(A)** hippocampal nNOS and **(B)** BDNF protein level. Correlations of pgWAT *Il6* mRNA expression with **(C)** hippocampal nNOS and **(D)** BDNF protein level in WT, *db/db* + vehicle, and *db/db* + TGF-β2 mice. The lines on the scatter plots show significant correlations (*n* = 4/group).

The hippocampal *Bdnf* mRNA expression was lower in the *db/db* + vehicle and *db/db* + TGF-β2 groups than in the WT + vehicle group (Figure 4A). The mRNA expression of the HFD-responsive and anxiety-related gene *Nos1a* was lower in *db/db* mice than in control mice, but TGF-β2 infusion had no effect on this (Figure 4A). Consistent with the mRNA data, TGF-β2 infusion did not affect the lower hippocampal BDNF and nNOS protein levels found in the *db/db* mice (Figures 4B,C). The expression of proBDNF in the hippocampus did not differ among the groups (Figures 4B,C). The hippocampal BDNF and nNOS protein levels negatively correlated with the pgWAT *Tnfa* mRNA expression (Figures 5A,B), but not with the *Il6* mRNA expression (Figures 5C,D).

## Discussion

Epidemiological studies have shown links between T2D and mood disorders (Fisher et al., 2008; McCrimmon et al., 2012; Cherbuin et al., 2015), and several studies have shown that peripheral and central inflammatory signaling may explain these links. Therefore, we aimed to determine whether the anti-inflammatory cytokine TGF-β2 would ameliorate obesity, glucose dyshomeostasis, and anxiety-like behaviors using a mouse model of T2D that was chronically infused with TGF-β2. The major findings of the study were as follows: 1) TGF-β2 ameliorated the abnormal expression of pro-inflammatory markers in adipose tissue, but had no effects on the glucose intolerance, anxiety-like behaviors, or low hippocampal BDNF and nNOS expression of *db/db* mice; and 2) the hippocampal BDNF and nNOS protein levels correlated with the expression of the pro-inflammatory gene *Tnfa*, but not *Il6*, in adipose tissue.

Contrary to our hypothesis, there were no marked effects of TGF-β2 infusion on the glucose tolerance of *db/db* mice (Figure 1). Although the effects of TGF-β2 in diabetes have been studied in the context of complications, such as retinopathy (Kita et al., 2007; Yang et al., 2010), its effects on diabetes and glucose metabolism are poorly understood. Previously, TGF-β2 treatment was found to improve the glucose tolerance and insulin sensitivity of HFD-fed obese mice (Takahashi et al., 2019), but these effects were not identified in the present study. The reason for this discrepancy is not readily apparent, but may be related to differences in the signaling pathways mediating glucose uptake in skeletal muscle in *db/db* and diet-induced obese mice (Halseth et al., 2002; Iglesias et al., 2002). Takahashi *et al.* demonstrated that TGF-β2 normalizes the infiltration of macrophages into the adipose tissue of obese mice, and similarly, we have demonstrated a TGF-β2-induced reduction in pro-inflammatory gene expression in the pgWAT of *db/db* mice (Figure 3A). Therefore, in severe obesity, such as that present in *db/db* mice, TGF-β2 treatment can reduce the degree of inflammation, but might be insufficient to improve glucose homeostasis. Ibuprofen, an anti-inflammatory drug, also has no effect on blood glucose concentration in a model of type 1 DM (Qiao et al., 2015) or on the circulating concentrations of pro-inflammatory markers, such as Il-6 and Il-1β, in *db/db* mice (Fourrier et al., 2019). Thus, it may be possible that the physiological effects of anti-inflammatory drugs are blunted in diabetic model rodents.

The number of closed arm entries and the total number of arm entries during EPM testing were significantly lower in *db/db* mice (Figure 2). In general, a reduction in the number of closed arm entries implies an amelioration of anxiety-like behaviors. However, the total number of entries was also low, which indicates lower locomotor activity, and it has been previously shown that rodents with diabetes move around less (Hussain et al., 2019). A previous methodological study showed that the total number of arm entries represents a useful index of anxiety-like behaviors (Komada et al., 2008). Thus, the present findings might imply that *db/db* mice have significant anxiety, similar to that described previously (Dinel et al., 2011; Fourrier et al., 2019). This conclusion is supported by the larger amount of time that *db/db* mice spent resting during EPM testing (Figure 2D). We found that hippocampal pro-inflammatory marker gene expression was not affected by the *db/db* phenotype or TGF-β2 treatment (Figure 3B). These findings suggest that central inflammation might not occur in *db/db* mice and might not contribute to their diabetes-related behavioral abnormalities.

Previously, it has been shown that hippocampal BDNF and nNOS affect cognitive function, including mood-related behaviors, in obesity (Molteni et al., 2002; Cai et al., 2016; Tomiga et al., 2019). In the hippocampus, because the selective inhibition of nNOS increases BDNF protein levels, nNOS is considered to be an upstream regulator of BDNF (Stanquini et al., 2018). Diabetes impairs hippocampal function (Stranahan et al., 2008). The present data suggest that BDNF and nNOS expression is low at both the mRNA and protein levels in the hippocampus of *db/db* mice. The low expression of BDNF in *db/db* mice is consistent with the results of previous studies of other mouse models of diabetes (Wosiski-Kuhn et al., 2018; Hussain et al., 2019).

BDNF is initially synthesized as a precursor, proBDNF, which is subsequently cleaved to form mature BDNF. Patients with major depressive disorders show low serum BDNF concentrations, while their serum proBDNF concentrations are high (Jiang et al., 2017). In a previous study, opposite trends were identified with respect to BDNF and proBDNF, and corticosterone was shown to increase hippocampal proBDNF expression, which negatively correlated with the number of open arm entries (Lin et al., 2022). However, we found that the proBDNF protein levels did not differ among the groups in the present study. Because the mature BDNF protein levels in the hippocampi of *db/db* mice were low, it is possible that the proteolytic cleavage of proBDNF is impaired in the CNS in severe obesity.

In the present study, the diabetes-related behavioral defects of the *db/db* mice were not ameliorated by the TGF-β2 treatment. Originally, we hypothesized that circulating TGF-β2 and endogenous *Tgfb2* expression represent potential therapeutic targets for mood regulation. Previously, it was reported that circulating TGF-β2, but not TGF-β1, can cross the intact blood-brain barrier (Kastin et al., 2003; McLennan et al., 2005). TGF-β2 has been shown to positively regulate synaptic plasticity and activate CREB in hippocampal neurons (Fukushima et al., 2007). In addition, hippocampal *Tgfb2* mRNA expression is upregulated by anxiolytic drugs (Lee et al., 2010) and downregulated by chronic stress (Grassi et al., 2017).

TGF-β2 expression is increased by potassium chloride-induced neuronal activity in cultured hippocampal neurons (Grassi et al., 2017). In addition, running exercise increases the circulating TGF-β2 concentration (Takahashi et al., 2019). nNOS and BDNF expression are regulated by the increase in neuronal activity that accompanies physical exercise (Zhang and Wong-Riley, 1999; Lu, 2003; Tomiga et al., 2021). Because physical exercise is commonly recommended for the management of T2D and the associated brain dysfunction, we aimed to determine whether TGF-β2 treatment would mimic the effect of exercise and its relationship with hippocampal nNOS and BDNF expression. However, consistent with the behavioral findings, TGF-β2 administration did not affect the low hippocampal nNOS and BDNF protein and mRNA expression in the *db/db* mice. In addition, because hippocampal *Tgfb2* mRNA was not affected by TGF-β2 administration, it may be inferred that exogenous TGF-β2 does not affect neuronal activation in the hippocampus in T2D. These findings suggest that circulating TGF-β2 might not affect the hippocampal regulation of mood in *db/db* mice.

The low hippocampal nNOS expression was contrary to our expectation. nNOS is the major NOS isoform in the adult brain (Bredt et al., 1994) and acts as a source of nitric oxide (NO) in the CNS. NO is considered to exert neuroprotective effects at low-to-moderate concentrations, but becomes neurotoxic at high concentrations (Abbott and Nahm, 2004). Zhang *et al.* showed that treatment with a selective serotonin reuptake inhibitor reduced hippocampal nNOS expression, and treatment with the nNOS-selective inhibitor 7-nitroindazole reduced anxiety-like behaviors (Zhang et al., 2010). Consistent with these findings, we previously reported that HFD-induced obesity is associated with high hippocampal nNOS expression (Tomiga et al., 2017), and that the expression level correlates with body mass and visceral adipose tissue accumulation (Tomiga et al., 2019). Physical activity, such as running, which is recognized to play a role in the maintenance of mental health, increases BDNF expression and reduces hippocampal nNOS expression (Tomiga et al., 2021). Therefore, the reason for the low nNOS expression in the hippocampi of the T2D *db/db* mice is not readily apparent. One possible mechanism might be nNOS protein turnover. We previously reported that hippocampal *Nos1* mRNA expression is low in the initial phase of HFD-induced obesity, prior to the upregulation of nNOS protein (Tomiga et al., 2019). Conversely, in the later phase of obesity, when hippocampal nNOS protein levels are high, Nos1 mRNA expression is low (Tomiga et al., 2019). Given these findings, when severe obesity develops, as in *db/db* mice, the low mRNA expression during the early phase of obesity might lead to low hippocampal nNOS protein levels. In addition, it has been shown that nNOS is degraded by the ubiquitin proteasome pathway (Bender et al., 2000). We can speculate that T2D might affect protein degradation pathways in the CNS and might contribute to these differences. In addition, in rodents with streptozotocin-induced type 1 diabetes, both low (Reagan and McEwen, 2002) and high (Guo et al., 2017) hippocampal nNOS levels have been identified. These findings suggest that hippocampal nNOS regulation differs according to the phase of obesity and might be not explained by differences in glucose homeostasis alone; therefore, further studies are needed.

Both the hippocampal BDNF and nNOS protein levels negatively correlated with pgWAT *Tnfa* mRNA expression, but not *Il6* mRNA expression (Figure 5). Several previous studies have shown that anxiety-like or depression-like behaviors in *db/db* mice are ameliorated by the intracerebroventricular (i.c.v.) or systemic administration of a TNF-α inhibitor (Fourrier et al., 2019; Alshammari et al., 2020). Similarly, the blockade of IL-6 receptor has an antidepressant effect in rodents with social defeat stress (Zhang et al., 2017). Interestingly, although the i. c.v. administration of TNF-α provokes an anxiogenic response, IL-6 has no effects on anxiety-like behaviors (Connor et al., 1998). These findings suggest that IL-6 itself might not affect central mood regulation directly. Given these findings, it is possible that peripherally-derived TNF-α in *db/db* mice might contribute to central mood regulation. Although our data does not confirm a causal relationship, the correlations between pgWAT *Tnfa* expression and the expression of hippocampal mood regulators, such as BDNF and nNOS, are consistent with this.

There were several limitations to the present study. First, we could not confirm that TGF-β2 had direct effects in the CNS in the *db/db* mice. McLennan *et al.* have shown that the entry of acutely administered TGF-β2 reaches a peak after 10 min in both the circulation and the brain, after which the concentration of TGF-β2 in the brain remains stable for at least 45 min (McLennan et al., 2005). We confirmed an anti-inflammatory effect of TGF-β2 in the pgWAT of *db/db* mice, which implies that the TGF-β2 entered the circulation at least. However, given that above findings, and although they provide indirect evidence, it can be inferred that TGF-β2 enters the CNS. Second, we did not include a TGF-β2-treated WT group. Therefore, it is unclear whether the effects of TGF-β2 are specific to the diabetic condition or whether it would have similar effects in normal animals. These limitations will be addressed in future studies.

In conclusion, in the present study, we have demonstrated behavioral defects, including in the resting time and movement speed of *db/db* mice. These behaviors and the low hippocampal BDNF and nNOS expression of *db/db* mice are not improved by chronic TGF-β2 infusion. In addition, the high body and adipose tissue masses and glucose intolerance of *db/db* mice are unaffected by chronic TGF-β2 infusion, and the adipose tissue pro-inflammatory marker gene expression of *db/db* mice is comparable to that of non-diabetic WT mice. These data suggest that although TGF-β2 ameliorates inflammation in T2D, it would not have a therapeutic effect in patients with T2D-induced mood alteration.

## Data Availability

The original contributions presented in the study are included in the article/supplementary material, further inquiries can be directed to the corresponding author.

## References

[B1] Abbott L. C. Nahm S. S. (2004). Neuronal nitric oxide synthase expression in cerebellar mutant mice. Cerebellum 3, 141–151. 10.1080/14734220410031927 15543804

[B2] Alshammari M. A. Khan M. R. Majid Mahmood H. Alshehri A. O. Alasmari F. F. Alqahtani F. M. (2020). Systemic TNF-α blockade attenuates anxiety and depressive-like behaviors in db/db mice through downregulation of inflammatory signaling in peripheral immune cells. Saudi Pharm. J. 28, 621–629. 10.1016/j.jsps.2020.04.001 32435144 PMC7229333

[B3] Bender A. T. Demady D. R. Osawa Y. (2000). Ubiquitination of neuronal nitric-oxide synthase *in vitro* and *in vivo* . J. Biol. Chem. 275, 17407–17411. 10.1074/jbc.M000155200 10751385

[B4] Bredt D. S. Snyder S. H. Hwang P. M. (1994). Transient nitric oxide synthase neurons in embryonic cerebral cortical plate, sensory ganglia, and olfactory epithelium. Neuron 13, 301–313. 10.1016/0896-6273(94)90348-4 7520252

[B5] Bruchas M. R. Land B. B. Lemos J. C. Chavkin C. (2009). CRF1-R activation of the dynorphin/kappa opioid system in the mouse basolateral amygdala mediates anxiety-like behavior. PLoS One 4, e8528–21. 10.1371/journal.pone.0008528 20052275 PMC2795205

[B6] Cai M. Wang H. Li J. J. Zhang Y. L. Xin L. Li F. (2016). The signaling mechanisms of hippocampal endoplasmic reticulum stress affecting neuronal plasticity-related protein levels in high fat diet-induced obese rats and the regulation of aerobic exercise. Brain Behav. Immun. 57, 347–359. 10.1016/j.bbi.2016.05.010 27189035

[B7] Cherbuin N. Fraser M. Sachdev P. Anstey K. J. (2015). Being overweight is associated with hippocampal atrophy : the PATH through life study. Int. J. Obes. 39, 1509–1514. 10.1038/ijo.2015.106 26041696

[B8] Connor T. J. Song C. Leonard B. E. Merali Z. Anisman H. (1998). An assessment of the effects of central interleukin-1beta, -2, -6, and tumor necrosis factor-alpha administration on some behavioural, neurochemical, endocrine and immune parameters in the rat. Neuroscience 84, 923–933. 10.1016/S0306-4522(97)00533-2 9579794

[B9] Conti A. C. Cryan J. F. Dalvi A. Lucki I. Blendy J. A. (2002). cAMP response element-binding protein is essential for the upregulation of brain-derived neurotrophic factor transcription, but not the behavioral or endocrine responses to antidepressant drugs. J. Neurosci. 22, 3262–3268. 10.1523/JNEUROSCI.22-08-03262.2002 11943827 PMC6757540

[B10] Coppack S. W. (2001). Pro-inflammatory cytokines and adipose tissue. Proc. Nutr. Soc. 60, 349–356. 10.1079/pns2001110 11681809

[B11] Dinel A. L. André C. Aubert A. Ferreira G. Layé S. Castanon N. (2011). Cognitive and emotional alterations are related to hippocampal inflammation in a mouse model of metabolic syndrome. PLoS One 6, e24325. 10.1371/journal.pone.0024325 21949705 PMC3174932

[B12] Fisher L. Skaff M. M. Mullan J. T. Arean P. Glasgow R. Masharani U. (2008). A longitudinal study of affective and anxiety disorders, depressive affect and diabetes distress in adults with type 2 diabetes. Diabet. Med. 25, 1096–1101. 10.1111/j.1464-5491.2008.02533.x 19183314 PMC2635496

[B13] Fourrier C. Bosch-Bouju C. Boursereau R. Sauvant J. Aubert A. Capuron L. (2019). Brain tumor necrosis factor-α mediates anxiety-like behavior in a mouse model of severe obesity. Brain Behav. Immun. 77, 25–36. 10.1016/j.bbi.2018.11.316 30508579

[B14] Fukushima T. Liu R.-Y. Byrne J. H. (2007). Transforming growth factor-beta2 modulates synaptic efficacy and plasticity and induces phosphorylation of CREB in hippocampal neurons. Hippocampus 17, 5–9. 10.1002/hipo.20243 17094084

[B15] Grassi D. Franz H. Vezzali R. Bovio P. Heidrich S. Dehghanian F. (2017). Neuronal activity, TGFβ-signaling and unpredictable chronic stress modulate transcription of Gadd45 family members and DNA methylation in the hippocampus. Cereb. Cortex 27, 4166–4181. 10.1093/cercor/bhx095 28444170

[B16] Guo F. Yue H. Wang L. Ding C. Wu L. Wu Y. (2017). Vitamin D supplement ameliorates hippocampal metabolism in diabetic rats. Biochem. Biophys. Res. Commun. 490, 239–246. 10.1016/j.bbrc.2017.06.028 28606476

[B17] Halseth A. E. Ensor N. J. White T. A. Ross S. A. Gulve E. A. (2002). Acute and chronic treatment of ob/ob and db/db mice with AICAR decreases blood glucose concentrations. Biochem. Biophys. Res. Commun. 294, 798–805. 10.1016/S0006-291X(02)00557-0 12061777

[B18] Hashimoto K. Shimizu E. Iyo M. (2004). Critical role of brain-derived neurotrophic factor in mood disorders. Brain Res. Brain Res. Rev. 45, 104–114. 10.1016/j.brainresrev.2004.02.003 15145621

[B19] Hussain Y. Jain S. K. Samaiya P. K. (2019). Short-term westernized (HFFD) diet fed in adolescent rats: Effect on glucose homeostasis, hippocampal insulin signaling, apoptosis and related cognitive and recognition memory function. Behav. Brain Res. 361, 113–121. 10.1016/j.bbr.2018.12.042 30584898

[B20] Iglesias M. A. Ye J. M. Frangioudakis G. Saha A. K. Tomas E. Ruderman N. B. (2002). AICAR administration causes an apparent enhancement of muscle and liver insulin action in insulin-resistant high-fat-fed rats. Diabetes 51, 2886–2894. 10.2337/diabetes.51.10.2886 12351423

[B21] Jiang H. Chen S. Li C. Lu N. Yue Y. Yin Y. (2017). The serum protein levels of the tPA-BDNF pathway are implicated in depression and antidepressant treatment. Transl. Psychiatry 7, e1079–5. 10.1038/tp.2017.43 28375203 PMC5416686

[B22] Kastin A. J. Akerstrom V. Pan W. (2003). Circulating TGF-beta1 does not cross the intact blood-brain barrier. J. Mol. Neurosci. 21, 43–48. 10.1385/JMN:21:1:43 14500993

[B23] Kita T. Hata Y. Kano K. Miura M. Nakao S. Noda Y. (2007). Transforming growth factor-beta2 and connective tissue growth factor in proliferative vitreoretinal diseases: possible involvement of hyalocytes and therapeutic potential of rho kinase inhibitor. Diabetes 56, 231–238. 10.2337/db06-0581 17192487

[B24] Komada M. Takao K. Miyakawa T. (2008). Elevated plus maze for mice. J. Vis. Exp. (22), e1088. 10.3791/1088 PMC2762911 19229173

[B25] Lavin D. N. Joesting J. J. Chiu G. S. Moon M. L. Meng J. Dilger R. N. (2009). Fasting induces an anti-inflammatory effect on the neuroimmune system which a high-fat diet prevents. Obesity 19, 1586–1594. 10.1038/oby.2011.73 PMC3695639 21527899

[B26] Lee J. H. Ko E. Kim Y. E. Min J. Y. Liu J. Kim Y. (2010). Gene expression profile analysis of genes in rat hippocampus from antidepressant treated rats using DNA microarray. BMC Neurosci. 11, 152. 10.1186/1471-2202-11-152 21118505 PMC3009642

[B27] Lin L. Herselman M. F. Zhou X. F. Bobrovskaya L. (2022). Effects of corticosterone on BDNF expression and mood behaviours in mice. Physiol. Behav. 247, 113721. 10.1016/j.physbeh.2022.113721 35074305

[B28] Liu W. Liu J. Xia J. Xue X. Wang H. Qi Z. (2017). Leptin receptor knockout-induced depression-like behaviors and attenuated antidepressant effects of exercise are associated with STAT3/SOCS3 signaling. Brain Behav. Immun. 61, 297–305. 10.1016/j.bbi.2017.01.001 28069387

[B29] Lu B. (2003). BDNF and activity-dependent synaptic modulation. Learn. Mem. 10, 86–98. 10.1101/lm.54603 12663747 PMC5479144

[B30] Lumeng C. N. Saltiel A. R. (2011). Inflammatory links between obesity and metabolic disease. J. Clin. Invest. 121, 2111–2117. 10.1172/JCI57132 21633179 PMC3104776

[B31] McCrimmon R. J. Ryan C. M. Frier B. M. (2012). Diabetes and cognitive dysfunction. Lancet 379, 2291–2299. 10.1016/S0140-6736(12)60360-2 22683129

[B32] McLennan I. S. Weible M. W. Hendry I. A. Koishi K. (2005). Transport of transforming growth factor-β2 across the blood-brain barrier. Neuropharmacology 48, 274–282. 10.1016/j.neuropharm.2004.10.005 15695166

[B33] Molteni R. Barnard R. J. Ying Z. Roberts C. K. Gómez-Pinilla F. (2002). A high-fat, refined sugar diet reduces hippocampal brain-derived neurotrophic factor, neuronal plasticity, and learning. Neuroscience 112, 803–814. 10.1016/S0306-4522(02)00123-9 12088740

[B34] Qiao W. Wang C. Chen B. Zhang F. Liu Y. Lu Q. (2015). Ibuprofen attenuates cardiac fibrosis in streptozotocin-induced diabetic rats. Cardiology 131, 97–106. 10.1159/000375362 25896805

[B35] Reagan L. P. McEwen B. S. (2002). Diabetes, but not stress, reduces neuronal nitric oxide synthase expression in rat hippocampus: Implications for hippocampal synaptic plasticity. Neuroreport 13, 1801–1804. 10.1097/00001756-200210070-00022 12395127

[B36] Semenkovich K. Brown M. E. Svrakic D. M. Lustman P. J. (2015).Depression in type 2 diabetes mellitus : Prevalence , impact , and treatment. Drugs 75, 577–587. 10.1007/s40265-015-0347-4 25851098

[B37] Shima T. Matsui T. Jesmin S. Okamoto M. Soya M. Inoue K. (2017). Moderate exercise ameliorates dysregulated hippocampal glycometabolism and memory function in a rat model of type 2 diabetes. Diabetologia 60, 597–606. 10.1007/s00125-016-4164-4 27928614

[B38] Stanquini L. A. Biojone C. Guimarães F. S. Joca S. R. (2018). Repeated treatment with nitric oxide synthase inhibitor attenuates learned helplessness development in rats and increases hippocampal BDNF expression. Acta Neuropsychiatr. 30, 127–136. 10.1017/neu.2017.28 29151391

[B39] Stranahan A. M. Arumugam T. V. Cutler R. G. Lee K. Egan J. M. Mattson M. P. (2008). Diabetes impairs hippocampal function through glucocorticoid-mediated effects on new and mature neurons. Nat. Neurosci. 11, 309–317. 10.1038/nn2055 18278039 PMC2927988

[B40] Takahashi H. Alves C. R. R. Stanford K. I. Middelbeek R. J. W. Nigro P. Ryan R. E. (2019). TGF-β2 is an exercise-induced adipokine that regulates glucose and fatty acid metabolism. Nat. Metab. 1, 291–303. 10.1038/s42255-018-0030-7 31032475 PMC6481955

[B41] Tomiga Y. Yoshimura S. Ito A. Nakashima S. Kawanaka K. Uehara Y. (2017). Exercise training rescues high fat diet-induced neuronal nitric oxide synthase expression in the hippocampus and cerebral cortex of mice. Nitric Oxide 66, 71–77. 10.1016/j.niox.2017.03.002 28302517

[B42] Tomiga Y. Yoshimura S. Ra S.-G. Takahashi Y. Goto R. Kugimoto I. (2019). Anxiety-like behaviors and hippocampal nNOS in response to diet-induced obesity combined with exercise. J. Physiol. Sci. 69, 711–722. 10.1007/s12576-019-00686-5 31124076 PMC10717450

[B43] Tomiga Y. Sakai K. Nakashima S. Uehara Y. Kawanaka K. Higaki Y. (2020). Effects of inosine monophosphate and exercise training on neuronal nitric oxide synthase in the mouse brain. Neurosci. Lett. 734, 135083. 10.1016/j.neulet.2020.135083 32479857

[B44] Tomiga Y. Sakai K. Ra S.-G. Kusano M. Ito A. Uehara Y. (2021). Short-term running exercise alters DNA methylation patterns in neuronal nitric oxide synthase and brain-derived neurotrophic factor genes in the mouse hippocampus and reduces anxiety-like behaviors. FASEB J. 35, e21767. 10.1096/fj.202100630R 34325488

[B45] Unsicker K. Flanders K. C. Cissel D. S. Lafyatis R. Sporn M. B. (1991). Transforming growth factor beta isoforms in the adult rat central and peripheral nervous system. Neuroscience 44, 613–625. 10.1016/0306-4522(91)90082-y 1754055

[B46] Wada T. Onogi Y. Kimura Y. Nakano T. Fusanobori H. Ishii Y. (2013). Cilostazol ameliorates systemic insulin resistance in diabetic db/db mice by suppressing chronic inflammation in adipose tissue via modulation of both adipocyte and macrophage functions. Eur. J. Pharmacol. 707, 120–129. 10.1016/j.ejphar.2013.03.016 23528355

[B47] Winkler G. Kiss S. Keszthelyi L. Sápi Z. Öry I. Salamon F. (2003). Expression of tumor necrosis factor (TNF)-α protein in the subcutaneous and visceral adipose tissue in correlation with adipocyte cell volume, serum TNF-α, soluble serum TNF-receptor-2 concentrations and C-peptide level. Eur. J. Endocrinol. 149, 129–135. 10.1530/eje.0.1490129 12887290

[B48] Wosiski-Kuhn M. Bota M. Snider C. A. Wilson S. P. Venkataraju K. U. Osten P. (2018). Hippocampal brain-derived neurotrophic factor determines recruitment of anatomically connected networks after stress in diabetic mice. Hippocampus 28, 900–912. 10.1002/hipo.23018 30098276 PMC6544163

[B49] Yang H. Huang Y. Chen X. Liu J. Lu Y. Bu L. (2010). The role of CTGF in the diabetic rat retina and its relationship with VEGF and TGF-β2, elucidated by treatment with CTGFsiRNA. Acta Ophthalmol. 88, 652–659. 10.1111/j.1755-3768.2009.01641.x 20039857

[B50] Yoshimura A. Wakabayashi Y. Mori T. (2010). Cellular and molecular basis for the regulation of inflammation by TGF-beta. J. Biochem. 147, 781–792. 10.1093/jb/mvq043 20410014 PMC2912031

[B51] Zhang C. Wong-Riley M. (1999). Expression and regulation of NMDA receptor subunit R1 and neuronal nitric oxide synthase in cortical neuronal cultures: Correlation with cytochrome oxidase. J. Neurocytol. 28, 525–539. 10.1023/A:1007053204929 10800203

[B52] Zhang J. Huang X.-Y. Ye M. Luo C. Wu H.-Y. Hu Y. (2010). Neuronal nitric oxide synthase alteration accounts for the role of 5-HT1A receptor in modulating anxiety-related behaviors. J. Neurosci. 30, 2433–2441. 10.1523/JNEUROSCI.5880-09.2010 20164327 PMC6634557

[B53] Zhang J. C. Yao W. Dong C. Yang C. Ren Q. Ma M. (2017). Blockade of interleukin-6 receptor in the periphery promotes rapid and sustained antidepressant actions: a possible role of gut-microbiota-brain axis. Transl. Psychiatry 7, e1138. 10.1038/tp.2017.112 28556833 PMC5534942

[B54] Zhang S. Y. Ji S. X. Bai X. M. Yuan F. Zhang L. H. Li J. (2019). L-3-n-butylphthalide attenuates cognitive deficits in db/db diabetic mice. Metab. Brain Dis. 34, 309–318. 10.1007/s11011-018-0356-6 30506335

